# Unusually Small Thermal Expansion of Ordered Perovskite Oxide CaCu_3_Ru_4_O_12_ with High Conductivity

**DOI:** 10.3390/ma11091650

**Published:** 2018-09-07

**Authors:** Akihiro Tsuruta, Katsuhiro Nomura, Masashi Mikami, Yoshiaki Kinemuchi, Ichiro Terasaki, Norimitsu Murayama, Woosuck Shin

**Affiliations:** 1 National Institute of Advanced Industrial Science and Technology (AIST), Shimo-Shidami, Moriyama-ku, Nagoya 463-8560, Japan; nomura-k@aist.go.jp (K.N.); m-mikami@aist.go.jp (M.M.); y.kinemuchi@aist.go.jp (Y.K.); terra@cc.nagoya-u.ac.jp (I.T.); w.shin@aist.go.jp (W.S.); 2 Department of Physics, Nagoya University, Furo-cho, Chuikusa-ku, Nagoya 464-8602, Japan; 3 National Institute of Advanced Industrial Science and Technology (AIST), 1-1-1 Higashi, Tsukuba 305-8565, Japan; n-murayama@aist.go.jp

**Keywords:** ceramics heater, conducting oxide, perovskite, thermal expansion

## Abstract

We measured the coefficient of thermal expansion (CTE) of conducting composite ceramics 30 vol.% CuO-mixed CaCu_3_Ru_4_O_12_ together with CaCu_3_Ru_4_O_12_ and CuO. Although conducting ceramics tend to show higher CTE values than insulators, and its CTE value does not match with other ceramic materials, the CTE of CaCu_3_Ru_4_O_12_ (7–9 × 10^−6^/K) was as small as those of insulators such as CuO (9 × 10^−6^/K), alumina (8 × 10^−6^/K), and other insulating perovskite oxides. We propose that the thermal expansion of CaCu_3_Ru_4_O_12_ was suppressed by the Cu-O bond at the A-site due to the Jahn–Teller effect. This unusually small CTE of CaCu_3_Ru_4_O_12_ compared to other conducting oxides plays a vital role enabling successful coating of 30 vol.% CuO-mixed CaCu_3_Ru_4_O_12_ thick films on alumina substrates, as demonstrated in our previous study.

## 1. Introduction

The coefficient of thermal expansion (CTE) and matching the thermal expansion between different materials are important factors when processing brittle ceramic materials that require high sintering temperatures. Currently, in order to use the heat resistance and functions of ceramic materials, devices containing heterogeneous ceramics with ceramic/ceramic or metal/ceramic interfaces have been actively developed using cofiring [1,2], printing [3,4], and coating [5,6,7,8,9,10] processes in various industrials fields, including energy [11,12,13], automotive [14,15], and healthcare [16,17]. Since the target ceramic materials are rarely well-sintered at the desired position when sintered with other materials, the thermal expansion needs to be tailored by optimizing the process, adding complementary materials, or controlling the composition of the ceramic [18,19]. In the case of devices using functional materials, such as perovskite oxides with CTE values often higher than those of other oxides, their thermal expansion behavior has been extensively investigated. When the perovskite composition is modified by the addition of other materials for thermal expansion matching, the properties of the original material may be degraded.

**Figure 1:**
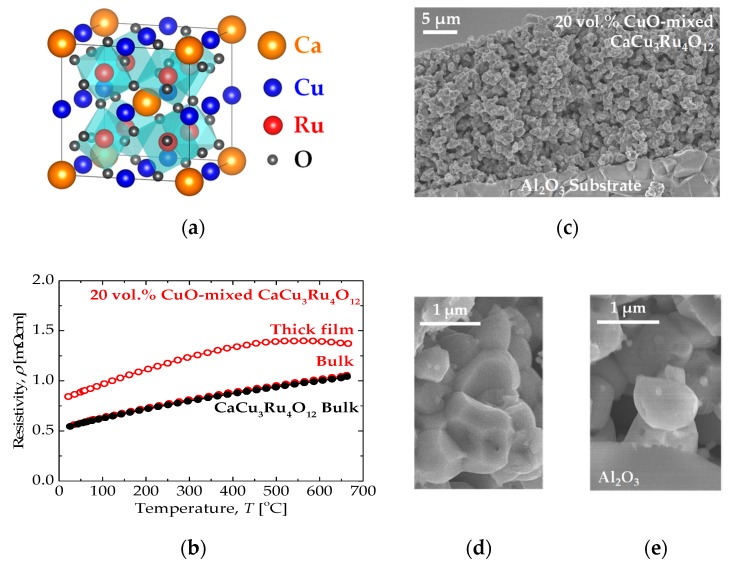
(**a**) Crystal structure of CaCu_3_Ru_4_O_12_. (**b**) Temperature dependence of the resistivity of a CaCu_3_Ru_4_O_12_ bulk, a 20 vol.% CuO-mixed CaCu_3_Ru_4_O_12_ bulk, and a 20 vol.% CuO-mixed CaCu_3_Ru_4_O_12_ thick film. (**c**) Cross-sectional SEM image of a 20 vol.% CuO-mixed CaCu_3_Ru_4_O_12_ thick film on an alumina substrate. (**d**) and (**e**) are magnified images of the film and the film–substrate interface, respectively, shown in (**c**).

We have focused on the conducting oxide CaCu_3_Ru_4_O_12_ [20,21,22,23,24] as an alternative conducting material to replace Pt in various high-temperature electrical devices, such as gas sensors [16] and solid oxide fuel cells [25], and have studied its physical properties and processing [26,27]. CaCu_3_Ru_4_O_12_ is an ordered perovskite oxide, the crystal structure of which is shown in the schematic diagram in Figure 1a. The resistivity is lower than 1 m·Ωcm, even at 500 °C, and the temperature dependence shows metallic behavior, which is rarely seen in oxides, as shown in Figure 1b (black plot). Although CaCu_3_Ru_4_O_12_ is difficult to sinter, we overcame this drawback by adding CuO as a sintering additive, which enabled the fabrication of dense bulks and thick films on alumina substrates [26]. The temperature dependences of the resistivity of 20 vol.% CuO-mixed CaCu_3_Ru_4_O_12_ bulk and thick film are shown in Figure 1b. The thick film showed resistivity as low as the bulk sample. Scanning Electron Microscope (SEM) images of the thick film are shown in Figure 1c–e, where CaCu_3_Ru_4_O_12_ grains were firmly bound to adjacent CaCu_3_Ru_4_O_12_ grains and to the alumina substrate without cracks and peeling. Recently, we tried to fabricate a SnO_2_ gas sensor using CuO-mixed CaCu_3_Ru_4_O_12_ thick films as electrodes and heater instead of Pt on an alumina substrate [28]. Our trial successfully showed similar sensing performance as the sensor using Pt. In addition, CuO-mixed CaCu_3_Ru_4_O_12_ thick film heaters on alumina substrates were robust against thermal shock and rapid thermal cycling.

We observed that CaCu_3_Ru_4_O_12_ and CuO can be easily compounded, and the composite thick film showed excellent sintering on an alumina substrate without any cracks. We further observed that the thick film heater showed excellent durability against rapid heat cycles. All these findings were truly unexpected for a conventional ceramic device. Hence, in this study, we investigated the thermal expansion of CaCu_3_Ru_4_O_12_, CuO, and CuO-mixed CaCu_3_Ru_4_O_12_ in order to clarify the reason for the excellent properties of our ceramic device. In addition, we compared the thermal expansion of CaCu_3_Ru_4_O_12_ with that of other perovskite oxides.

## 2. Experimental

CaCu_3_Ru_4_O_12_ was prepared via a solid-state reaction [22,26,29]. Stoichiometric mixtures of CaCO_3_, CuO, and RuO_2_ were pressed into pellets and calcined in air at 1000 °C for 48 h. The pellets were covered by a mixture of excess CaCO_3_, CuO, and RuO_2_ powders to prevent Ru sublimation during sintering and subsequent deviations from the desired composition. The CaCu_3_Ru_4_O_12_ powder was obtained via mechanical grinding and ball-milling of the calcined pellets. 

The CaCu_3_Ru_4_O_12_ powder was then mixed with CuO powder (as a sintering additive), then pressed into a pellet and sintered at 1000 °C for 48 h in air. The CuO volume fraction in the bulk was 30 vol.% corresponding to 29.5 wt.%. The volume fraction was calculated using the molecular weights and lattice constants of each material. A CuO bulk sample was obtained by calcining a pressed pellet of CuO powder under the same conditions as the 30 vol.% CuO-mixed CaCu_3_Ru_4_O_12_ bulk sample. The relative densities of the 30 vol.% CuO-mixed CaCu_3_Ru_4_O_12_ and CuO bulk samples were 73% and 92%, respectively.

X-ray diffraction (XRD) of the CaCu_3_Ru_4_O_12_ powder was performed using a standard diffractometer with parallel-beam optics of Cu K*α* radiation in the 2*θ*-*θ* scan mode (X’Pert Pro MPD, Malvern Panalytical, Malvern, UK) at 25, 100, 200, 300, 400, 500, 600, 700, 800, and 900 °C using a reactor chamber (XRK900, Anton Paar, Graz, Austria) in air [30]. The XRD patterns at all temperatures were analyzed using the Rietveld method [31] and we calculated the lattice constants using the reported space group and crystal structural parameters as the initial value [32]. The CTEs of 30 vol.% CuO-mixed CaCu_3_Ru_4_O_12_ and CuO bulk samples were measured in air using a thermomechanical analyzer (TMA; Thermo Plus EVO2, Rigaku, Tokyo, Japan).

## 3. Results and Discussion

**Figure 2:**
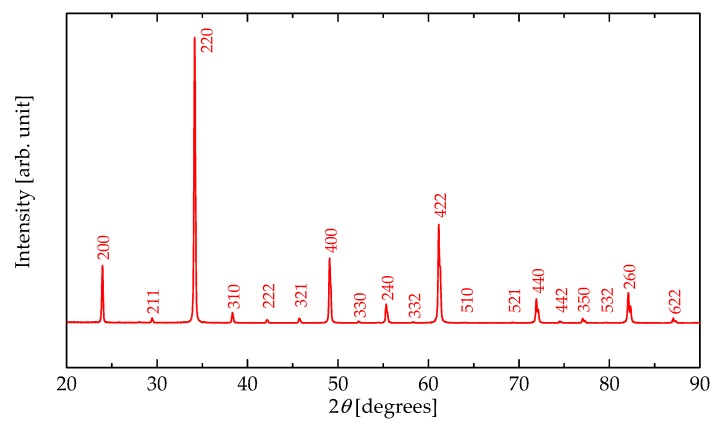
X-ray diffraction (XRD) (CuKα) pattern of CaCu_3_Ru_4_O_12_ powder measured at 25 °C.

**Figure 3:**
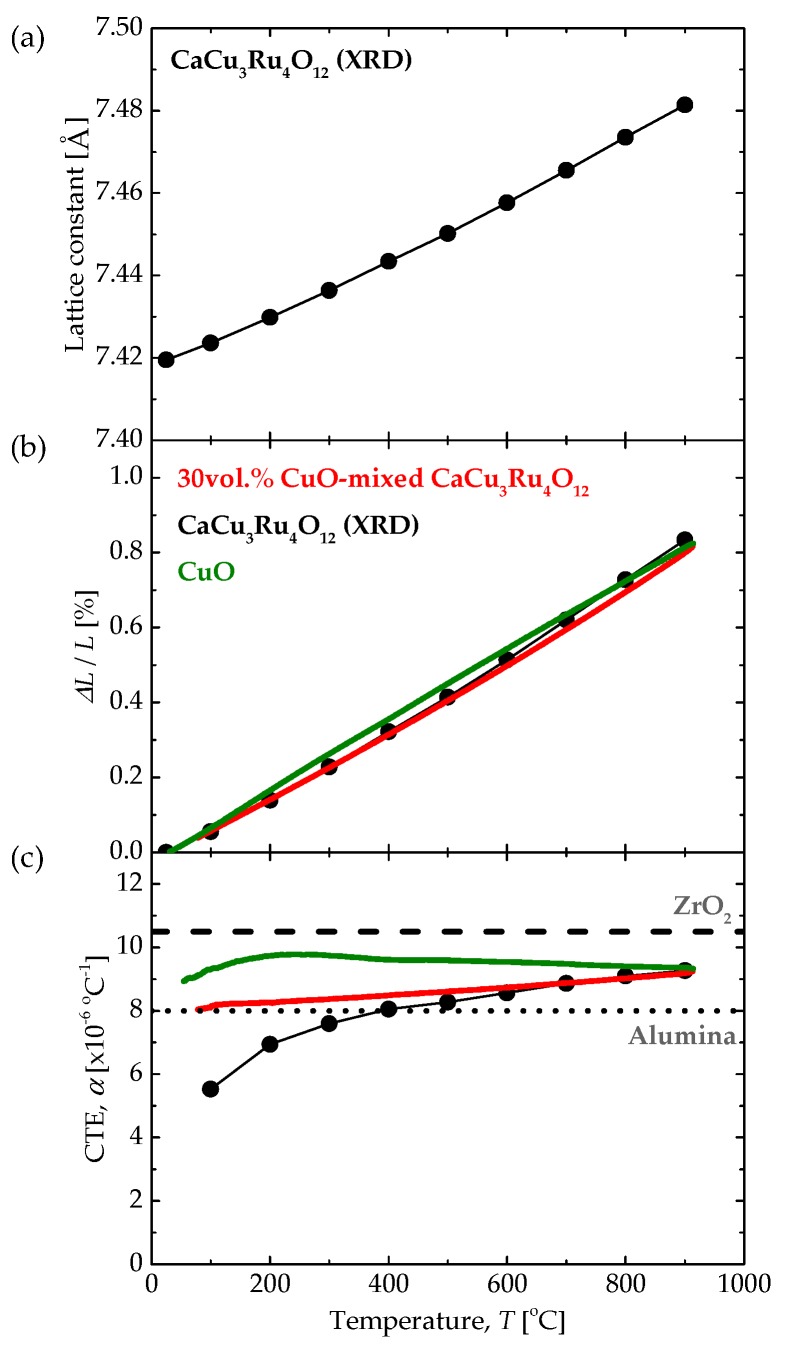
(**a**) Lattice constant of CaCu_3_Ru_4_O_12_ as a function of temperature. (**b**) Temperature dependence of *ΔL*/*L*_25_ and (**c**) temperature dependence of the CTE of the 30 vol.% CuO-mixed CaCu_3_Ru_4_O_12_ bulk, CuO bulk, and CaCu_3_Ru_4_O_12_ samples. Data for the 30 vol.% CuO-mixed CaCu_3_Ru_4_O_12_ bulk and CuO bulk were measured using a thermomechanical analyzer (TMA), while those for CaCu_3_Ru_4_O_12_ were calculated from powder XRD.

Figure 2 shows the XRD pattern for CaCu_3_Ru_4_O_12_ powder measured at 25 °C. All peaks were well-indexed to those of CaCu_3_Ru_4_O_12_ [32] and no heterogeneous or impurity phases were identified. Figure 3a shows the lattice constant of CaCu_3_Ru_4_O_12_ calculated from powder XRD, plotted as a function of temperature. CaCu_3_Ru_4_O_12_ belongs to a large family of ordered perovskites described by the general formula AC_3_B_4_O_12_, and can be considered as a fourfold superstructure of the ABO_3_ perovskite shown in Figure 1a. The lattice constant increased with increasing temperature, indicating positive thermal expansion, similar to other conventional oxide materials. The plot represents the lattice constant *a* for the cubic structure, from which the relative thermal expansion and CTE values were evaluated. 

Considering the potential applications of the perovskite, the thermal expansion should be evaluated using TMA. However, here the CTE of CaCu_3_Ru_4_O_12_ had to be calculated from the lattice constant because CaCu_3_Ru_4_O_12_ could not be fully sintered. Figure 3b shows the thermal expansion relative to the value at 25 °C (*ΔL*/*L*_25_) for the 30 vol.% CuO-mixed CaCu_3_Ru_4_O_12_ and CuO bulk samples measured using TMA, together with that of CaCu_3_Ru_4_O_12_ calculated from the data shown in Figure 3a. The thermal-expansion curves of CaCu_3_Ru_4_O_12_ and CuO were nearly identical between 25 °C and 900 °C. Hence, we concluded that the addition of 30 vol.% CuO to CaCu_3_Ru_4_O_12_ did not significantly affect thermal-expansion behavior, although a detailed analysis was not performed [33]. This good thermal-expansion match is the reason that no serious cracks or exfoliation at the interface between CaCu_3_Ru_4_O_12_ and CuO were observed in the composite material; this supports the results of our previous study where we successfully used CuO as a sintering additive for CaCu_3_Ru_4_O_12_ [26].

Figure 3c shows the temperature dependence of the CTE of the 30 vol.% CuO-mixed CaCu_3_Ru_4_O_12_ bulk, CuO bulk, and CaCu_3_Ru_4_O_12_ samples. The dotted and broken lines show the CTE at 400 °C of alumina and ZrO_2_, respectively, for comparison, as they are widely used ceramic substrate materials. Although the *ΔL*/*L*_25_ of the three materials were almost the same at all temperatures, the CTEs, which are generally calculated as the differential value of *ΔL*/*L*_25_, were slightly different, especially around room temperature. The CTE curve of the 30 vol.% CuO-mixed CaCu_3_Ru_4_O_12_ bulk sample was located between those of CuO bulk and CaCu_3_Ru_4_O_12_ at all temperatures, showing an acceptable average value. The 30 vol.% CuO-mixed CaCu_3_Ru_4_O_12_ bulk sample showed almost the same CTE value as alumina. Thus, in our previous study, the CuO-mixed CaCu_3_Ru_4_O_12_ thick film was successfully coated and sintered on alumina substrates without cracking or peering due to this good CTE match.

Inorganic materials have various favorable properties, including conductivity, dielectricity, and magnetism. The CTE (*α*) depends on such properties, as follows: [34]

*α* = *α*_vib_ + *α*_elec_ + *α*_mag_ + *α*_fe_ + *α*_vac_ (1)

where *α*_vib_, *α*_elec_, *α*_mag_, *α*_fe_, and *α*_vac_ correspond to the CTE due to vibrational, electronic, magnetic, ferroelectric contribution, and vacancy formation, respectively. This formula is derived considering that the CTE is the second derivative of the Gibbs energy, where functional materials have higher energies than materials without these properties. Therefore, *α* of a simple insulator should be the lowest among ceramic oxide materials, except for ZrW_2_O_8_ [35] and LaCu_3_Fe_4_O_12_ [36], which show negative thermal expansion due to peculiar mechanisms, such as lattice bending and valence transitions. Since CaCu_3_Ru_4_O_12_ shows particularly high electrical conductivity compared to other perovskite oxides, its *α*_elec_ and *α* values are expected to be larger than those of other materials. 

Let us compare CaCu_3_Ru_4_O_12_ with other ABO_3_-type oxides; Table 1 shows the CTE, conducting behavior, B-site cation, electron orbital of the B-site cation, and the number of d-electron of various oxides. Here, we focus on simple ABO_3_-type oxides in order to simplify the comparison and discussion. The CTE values were taken directly from the references, or calculated from the temperature dependence of the lattice volume. In this paper, the materials are classified as conductor or insulator using 10 Ωcm of resistivity at room temperature as a threshold. As expected, the conductors Sr_0.8_La_0.2_TiO_3_, SrRuO_3_, La_0.6_Sr_0.4_Fe_0.2_Co_0.8_O_3-*x*_, SrCoO_3_, LaCoO_3_, and LaCo_0.5_Ni_0.5_O_3_ show larger CTE values than materials with insulating, ferroelectric, or dielectric properties. However, the CTE of CaCu_3_Ru_4_O_12_ is remarkably small compared to other conductors. La_0.6_Sr_0.4_Fe_0.2_Co_0.8_O_3−*x*_, SrCoO_3_, and LaCoO_3_ are used as cathode materials in solid oxide fuel cells, and many oxygen vacancies are generated at high temperature; hence, *α*_elec._ and *α*_vac._ strongly contribute to *α* in these materials. 

**Figure 4:**
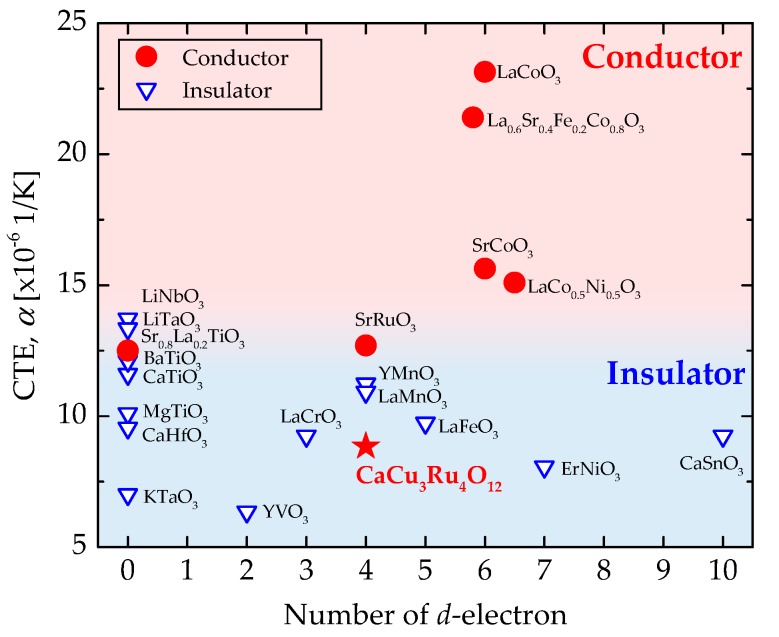
CTE of various perovskite oxides plotted as a function of the number of d-electrons.

The conduction behavior of the perovskite oxides correlates with the number of d-electrons in the B-site cation. Hence, CTE values are plotted as a function of the number of d-electrons in Figure 4, where the conduction behavior of each material is represented by symbols. The CTE values of all insulators are located in the lower part of the figure, consistent with the theory expressed by Equation (1). Although the ferroelectric materials, such as BaTiO_3_, LiTaO_3_ and LiNbO_3_, are electrically insulating, they show relatively large CTE values compared to the other insulators because of structural deformations due to polarization. Almost all conductors show CTE values larger than 10 × 10^−6^/K, where that of CaCu_3_Ru_4_O_12_ is surprisingly small, despite of its high electrical conductivity. CaCu_3_Ru_4_O_12_ and SrRuO_3_ are closely related materials; they both show high conductivity and have Ru occupying the B-site of perovskite. However, the CTE values of these materials are significantly different, where CaCu_3_Ru_4_O_12_ has a smaller value than the simple SrRuO_3_ perovskite. The structural difference between those materials is that Ca and Cu are ordered at the A-site of the perovskite structure in CaCu_3_Ru_4_O_12_. Cu does not usually occupy the A-site because Cu^2+^ normally appears in 6 coordination. However, Cu in CaCu_3_Ru_4_O_12_ is stabilized at the A-site owing to the Jahn–Teller effect. CuO octahedra extend in the *z*-axis direction with separation of the degenerated e_g_ orbital, while Cu^2+^ acts as a large cation and can occupy the A-site. Cu atoms occupy 75% of the A-site in CaCu_3_Ru_4_O_12_, and the Cu-O bond, which is stabilized at lower energy via separation of the degenerated e_g_ orbital, would be stronger than other simple bonds such as Ca-O and Ru-O. If this Cu-O bond is predominant in the thermal expansion of the CaCu_3_Ru_4_O_12_ lattice, the thermal expansion will be similar to that of CuO, as shown in Figure 3b. Such a contribution of the A-site cation to the thermal expansion is a novel feature of ordered perovskite and is a great advantage in material design and applications.

**Figure 5:**
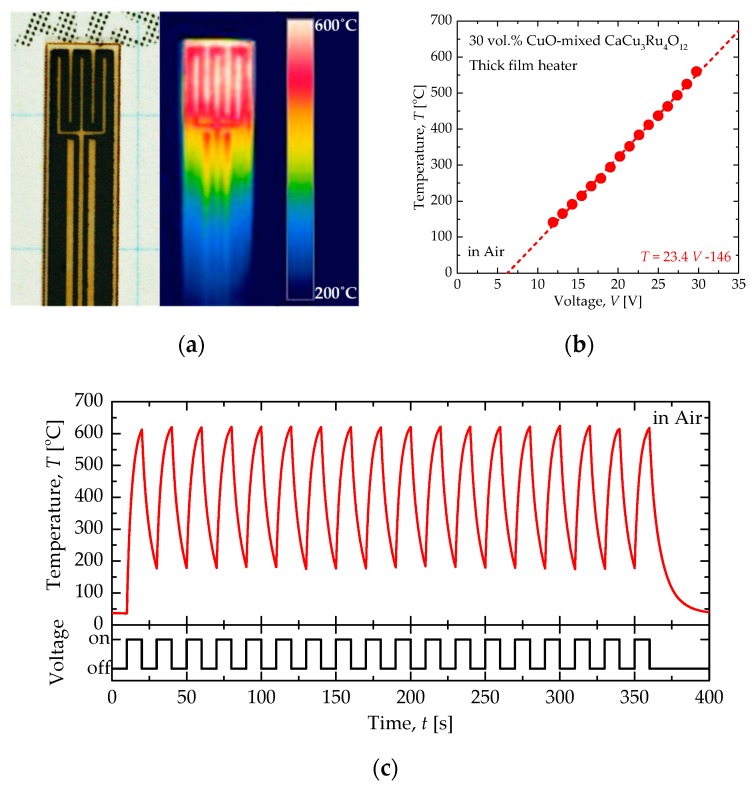
(**a**) Photograph of the 30 vol.% CuO-mixed CaCu_3_Ru_4_O_12_ thick-film heater on the alumina substrate and a thermal-camera image with an applied voltage of 32 V DC. (**b**) Temperature (*T*), defined as the maximum temperature over the meandering heater pattern, as a function of the applied voltage. (**c**) Temperature of the heater under cyclic 32.0 V pulses with a width of 10 s and cycle time of 20 s. All experiments were performed in air.

Finally, we show the heater characteristics of a 30 vol.% CuO-mixed CaCu_3_Ru_4_O_12_ thick-film heater on an alumina substrate realized by the unusually small thermal expansion of CaCu_3_Ru_4_O_12_ and CTE matching between CaCu_3_Ru_4_O_12_ and alumina. The thick-film heater was fabricated on an alumina substrate (3.0 × 25 × 0.3 mm) by screen-printing a paste of CuO and CaCu_3_Ru_4_O_12_ powders mixed in a suitable vehicle. Figure 5a shows a photograph and a thermal-camera image while 32 V DC voltage was applied to the 30 vol.% CuO-mixed CaCu_3_Ru_4_O_12_ thick-film heater. The meandering heater pattern generates heat up to 600 °C due to Joule heating. Figure 5b shows the temperature (*T*), defined as the maximum temperature over the heater pattern in air as a function of the applied voltage. The temperature increased linearly with applied voltage, expressed as *T* = 23.4 *V* − 146 in units of degrees Celsius; this linear *V*-*T* characteristic indicates good temperature controllability of the heater. The temperature of the heater under 32.0 V pulses, with a width of 10 s and a cycle length of 20 s, is shown in Figure 5c. The temperature increased immediately from room temperature when the voltage was applied, reaching 600 °C within 9 s. The heater temperature followed the cyclic voltage pulses without degradation, and the performance was maintained after many cycles. It is clear from these results that the 30 vol.% CuO-mixed CaCu_3_Ru_4_O_12_ thick-film heater on an alumina substrate is surprisingly robust and is thought to be reliable enough to be used as a substitute for Pt as a conducting material for various electrical devices.

## 4. Conclusions

We measured the CTE of 30 vol.% CuO-mixed CaCu_3_Ru_4_O_12_ and CuO bulk samples using TMA and calculated the CTE of CaCu_3_Ru_4_O_12_ from the lattice constant determined using high-temperature XRD measurements. The *ΔL*/*L*_25_ of these materials were nearly identical between 25 °C and 900 °C; we measured the values for CaCu_3_Ru_4_O_12_, CuO, and 30 vol.% CuO-mixed CaCu_3_Ru_4_O_12_ of 8.27 × 10^−6^ K^−1^, 9.59 × 10^−6^ K^−1^, and 8.60 × 10^−6^ K^−1^, respectively, at 500 °C. These values were similar to those of alumina (8.0 × 10^−6^ K^−1^). The CTE matches between CaCu_3_Ru_4_O_12_/CuO and 30 vol.% CuO-mixed CaCu_3_Ru_4_O_12_/alumina explain the successful compounding and coating of these material pairs demonstrated in our previous study. The CTE of CaCu_3_Ru_4_O_12_ is smaller than that of other conducting perovskite oxides, and close to the values for insulating perovskite oxides. The unusually small CTE of CaCu_3_Ru_4_O_12_ is thought to be due to the influence of Cu occupying the A-site of the perovskite via the Jahn–Teller effect. Such a contribution of the A-site cation in the ordered perovskite material to the thermal expansion offers a novel and superior advantage in material design and applications.

## Figures and Tables

**Table 1:** CTE, conduction behavior, B-site cation, electron orbital of B-site cation, and number of d-electron of various perovskite oxide.

Material		CTE (×10^−6^/K)	Conduction Behavior	B-Site Cation	Electron Orbital of B-Site Cation	Number of D-Electron
MgTiO_3_	[34]	10.1	Insulator	Ti^4+^	3d^0^	0
CaTiO_3_	[34]	11.6	Insulator	Ti^4+^	3d^0^	0
BaTiO_3_	[34]	12.1	Insulator	Ti^4+^	3d^0^	0
Sr_0.8_La_0.2_TiO_3_	[37]	12.5	Conductor	Ti^4+^	3d^0^	0
LiNbO_3_	[34]	13.7	Insulator	Nb^5+^	4d^0^	0
CaHfO_3_	[34]	9.6	Insulator	Hf^4+^	5d^0^	0
LiTaO_3_	[34]	13.3	Insulator	Ta^5+^	5d^0^	0
KTaO_3_	[34]	7.01	Insulator	Ta^5+^	5d^0^	0
YVO_3_	[38]	6.4	Insulator	V^3+^	3d^2^	2
LaCrO_3_	[34]	9.2	Insulator	Cr^3+^	3d^3^	3
YMnO_3_	[34]	11.2	Insulator	Mn^3+^	3d^4^	4
LaMnO_3_	[34]	10.9	Insulator	Mn^3+^	3d^4^	4
SrRuO_3_	[39]	12.7	Conductor	Ru^4+^	4d^4^	4
CaCu_3_Ru_4_O_12_		8.9	Conductor	Ru^4+^	4d^4^	4
LaFeO_3_	[34]	9.7	Insulator	Fe^3+^	3d^5^	5
La_0.6_S_r0.4_Fe_0.2_Co_0.8_O_3−*x*_	[19]	21.4	Conductor	Fe^3+^, Co^3+^	3d^5^, 3d^6^	5.8
SrCoO_3_	[34]	15.6	Conductor	Co^4+^	3d^5^	5
LaCoO_3_	[34]	23.1	Conductor	Co^3+^	3d^6^	6
LaCo_0.5_Ni_0.5_O_3_	[40]	15.1	Conductor	Co^3+^, Ni^3+^	3d^6^, 3d^7^	6.5
ErNiO_3_	[41]	8.1	Insulator	Ni^3+^	3d^7^	7
CaSnO_3_	[34]	9.2	Insulator	Sn^4+^	4d^10^	10

The CTE values are quoted at 500 °C; for the datasets where this value was not stated, the data were linearly extrapolated to 500 °C.
